# Avian influenza A(H5) virus circulation in live bird markets in Vietnam, 2017–2022

**DOI:** 10.1111/irv.13245

**Published:** 2023-12-27

**Authors:** Diep T. Nguyen, Kelsey M. Sumner, Thoa T. M. Nguyen, Minh Q. Phan, Tien M. Hoang, Chuong D. Vo, Tho D. Nguyen, Phuong T. Nguyen, Genyan Yang, Yunho Jang, Joyce Jones, Sonja J. Olsen, Philip L. Gould, Long V. Nguyen, Charles Todd Davis

**Affiliations:** ^1^ Department of Animal Health Hanoi Vietnam; ^2^ Influenza Division, National Center for Immunizations and Respiratory Disease Centers for Disease Control and Prevention Atlanta Georgia USA; ^3^ Epidemic Intelligence Service Centers for Disease Control and Prevention Atlanta Georgia USA; ^4^ National Center for Veterinary Diagnosis Department of Animal Health Hanoi Vietnam; ^5^ Regional Animal Health Officer Number 6 Department of Animal Health Ho Chi Minh City Vietnam

**Keywords:** avian influenza, live bird markets, poultry, surveillance, Vietnam

## Abstract

**Background:**

Highly pathogenic avian influenza A(H5) human infections are a global concern, with many A(H5) human cases detected in Vietnam, including a case in October 2022. Using avian influenza virus surveillance from March 2017–September 2022, we described the percent of pooled samples that were positive for avian influenza A, A(H5), A(H5N1), A(H5N6), and A(H5N8) viruses in live bird markets (LBMs) in Vietnam.

**Methods:**

Monthly at each LBM, 30 poultry oropharyngeal swab specimens and five environmental samples were collected. Samples were pooled in groups of five and tested for influenza A, A(H5), A(H5N1), A(H5N6), and A(H5N8) viruses by real‐time reverse‐transcription polymerase chain reaction. Trends in the percent of pooled samples that were positive for avian influenza were summarized by LBM characteristics and time and compared with the number of passively detected avian influenza outbreaks using Spearman's rank correlation.

**Results:**

A total of 25,774 pooled samples were collected through active surveillance at 167 LBMs in 24 provinces; 36.9% of pooled samples were positive for influenza A, 3.6% A(H5), 1.9% A(H5N1), 1.1% A(H5N6), and 0.2% A(H5N8). Influenza A(H5) viruses were identified January–December and at least once in 91.7% of sampled provinces. In 246 A(H5) outbreaks in poultry; 20.3% were influenza A(H5N1), 60.2% A(H5N6), and 19.5% A(H5N8); outbreaks did not correlate with active surveillance.

**Conclusions:**

In Vietnam, influenza A(H5) viruses were detected by active surveillance in LBMs year‐round and in most provinces sampled. In addition to outbreak reporting, active surveillance for A(H5) viruses in settings with high potential for animal‐to‐human spillover can provide situational awareness.

## 1 INTRODUCTION

From 2003 to 2022, 127 infections and 64 deaths with highly pathogenic avian influenza (HPAI) A(H5) viruses have been detected in individuals living in Vietnam^1^; this includes a case detected in October 2022, amidst a global increase of A(H5) virus infections in wild birds and poultry.^1^ In 2001, influenza A(H5N1) was first detected in Vietnam by identification of the virus in domestic poultry and anti‐A(H5N1) antibodies in humans, although the first human illnesses were not reported until 2003.^2^, ^3^, ^4^ Other A(H5) virus subtypes were detected in the country more recently, with influenza A(H5N6) virus first identified in domestic poultry in 2014 and influenza A(H5N8) virus in poultry in June 2021. However, no human cases of influenza A(H5N6) have been reported in Vietnam as of June 2023.^5^

Vietnam experiences repeated introductions of influenza A(H5) viruses possibly from migratory wild bird patterns or trade with bordering countries. Live bird markets (LBMs) are a common place where individuals trade large volumes of live poultry. Due to their variable infrastructure and biosecurity practices,^6^ LBMs are a major focal point for environmental contamination and poultry‐to‐poultry transmission of A(H5) viruses, resulting in an interface where humans may be exposed to avian influenza viruses.^6^, ^7^, ^8^ Influenza A(H5) virus surveillance is conducted at LBMs to monitor virus circulation and possible emergence of new strains and to provide a basis for avian influenza poultry vaccine recommendations at the national and provincial level; this surveillance is important because outbreak detections do not always correlate with routine surveillance activities.^9^

In a collaboration between the Vietnamese Ministry of Agriculture and Rural Development, Department of Animal Health (DAH), and the US Centers for Disease Control and Prevention (CDC), avian influenza surveillance has been conducted in provinces in Vietnam with active LBMs since 2013. Using avian influenza surveillance data from 2017–2022, we describe the geographic distribution and percent positivity of avian influenza A(H5N1), A(H5N6), and A(H5N8) viruses detected through active LBM surveillance and passive outbreak detection in Vietnam.

## 2 METHODS

Avian influenza A(H5) virus detections were captured through two approaches: (i) passive surveillance of influenza A(H5N1), A(H5N6), and A(H5N8) outbreaks reported to the Vietnamese DAH and (ii) active surveillance of influenza A, A(H5), A(H5N1), A(H5N6), and A(H5N8) viruses conducted in LBMs across 24 provinces in Vietnam. Vietnam's national active surveillance for avian influenza has been previously described.^6^, ^7^, ^9^

### 2.1 Passive detection of animal outbreaks

Outbreaks of influenza in poultry were identified and reported by local veterinary staff based on poultry die‐offs or illnesses in flocks. The Sub‐Department of Animal Health staff conducted outbreak investigations in coordination with the Regional Animal Health Office (RAHO). All outbreaks of influenza A(H5N1), A(H5N6), and A(H5N8) viruses were confirmed by real‐time reverse‐transcription polymerase chain reaction (rRT‐PCR) at a RAHO or National Center for Veterinary Diagnostics (NCVD) laboratory. All outbreak information was reported to the DAH for management and support. Confirmed outbreaks of A(H5N1), A(H5N6), and A(H5N8) viruses were reported to the World Organization for Animal Health. In the event of a confirmed outbreak of one of these viruses, all infected poultry and other unvaccinated poultry flocks with close contact to infected poultry were disposed of by the local veterinary team. Disposal was done as soon as possible at the farm or household to reduce transmission.

### 2.2 Selection of surveillance sites for active surveillance

An active surveillance program for avian influenza in LBMs was developed by the Vietnamese DAH in collaboration with US CDC. During the nearly 6‐year period from March 2017 to September 2022, LBMs across Vietnam were sampled using a stratified, multi‐stage cluster sampling design. Provinces for avian influenza surveillance were selected based on the following criteria: (i) frequency of annual reported HPAI outbreaks; (ii) poultry population density; (iii) level of poultry consumption; (iv) presence of cross‐border trading with neighboring countries; and (v) a strong commitment to conduct surveillance. In each province, four LBMs were chosen for regular surveillance; this included traditional‐ and non‐traditional LBMs. Traditional LBMs are larger‐scale markets or poultry gathering points that operate daily and receive poultry from outside the province, and therefore include representation of influenza A(H5) viruses circulating outside the province. Non‐traditional LBMs primarily receive local birds from the district or province and are more representative of local circulation of A(H5) viruses. To be selected for active surveillance for avian influenza, each LBM was required to regularly have at least six poultry vendors.

### 2.3 Sampling of birds and the environment for active surveillance

Monthly at each LBM, oropharyngeal swab specimens were collected by convenience sampling from a total of 30 poultry at six poultry vendors (five poultry per vendor). Environmental samples were also collected from poultry feces or water containers from five different places within the LBM. Oropharyngeal specimens were pooled together in groups of five by vendor, and environmental samples pooled in groups of five by LBM. Pooled samples were stored in a single tube with 2 mL viral transport medium. Samples were kept in a cold box and transported to a RAHO or NCVD laboratory within 48 hours after sampling. Seventy‐two hours after collection, untested samples were stored in −80°C freezers until further processing.

### 2.4 Laboratory testing of pooled samples collected in active surveillance

Viral RNA was extracted from pooled samples using Qiagen Viral RNA Extraction kits (Qiagen, Hilden, Germany) and then screened by rRT‐PCR to detect the influenza A virus matrix (M) gene.^10^ All influenza A virus positive samples were subsequently tested for the A(H5) subtype. Pooled samples positive for A(H5) viral RNA were then tested in parallel for influenza A N1, N6, and N8 neuraminidase gene targets. A cycle threshold value ≤35 indicated positivity for influenza A (M gene), H5, N1, N6, and/or N8 in rRT‐PCR assays.

### 2.5 Statistical analysis

Avian influenza A(H5) viruses detected actively at LBMs and passively by outbreaks were summarized at the province level. For poultry and environmental samples collected through active surveillance for avian influenza at LBMs, we calculated the percent positivity for influenza A, A(H5), A(H5N1), A(H5N6) and A(H5N8) viruses over time, poultry species, and market type. Percent positivity was defined as the number of positive poultry or environmental sample pools divided by the total number of sample pools tested at each level. Ninety‐five percent confidence intervals for each estimate were calculated using the “epiR” package implemented in R.^11^, ^12^ Within each province, influenza A(H5N1), A(H5N6), and A(H5N8) percent positivity was mapped for each year of active surveillance for avian influenza using the “ggmap” and “maptools” packages in R.^12^, ^13^, ^14^

The temporal distribution of percent positivity of A(H5), A(H5N1), A(H5N6), and A(H5N8) viruses detected in LBMs and the number of passively reported outbreaks were plotted over the study period. Correlation between monthly influenza A(H5N1), A(H5N6), and A(H5N8) percent positivity and number of reported outbreaks detected was calculated using Spearman's rank correlation; the correlation analysis was repeated using a 30‐day lag for LBM detections as a sensitivity analysis. For active surveillance, the monthly mean A(H5) percent positivity was calculated by averaging across all 6 years and 95% confidence intervals estimated by bootstrapping around the mean estimate 999 times using the “DescTools” package in R.^15^ All analyses were conducted in R version 4.2.2.^12^

## 3 RESULTS

Active surveillance for avian influenza was conducted almost monthly in either 11 or 13 provinces (depending on the year) in Vietnam from 2017–2021 and expanded to 22 provinces in 2022. During the nearly six‐year period from March 2017 to September 2022, 128,870 specimens were collected from poultry or their environment at a total of 167 LBMs in 24 provinces (out of 63 total provinces in the country) (Table 1). Individual poultry specimens and environmental samples were pooled into groups of five to create 25,774 pooled samples. Influenza A(H5) virus was identified at least once in 91.7% (95% confidence interval [CI]: 74.2–97.7) of provinces sampled and 54.9% (95% CI: 47.2–62.3) of LBMs sampled (Table S1).

**TABLE 1:** Distribution of samples collected through active surveillance for influenza A(H5) viruses in live bird markets and passive A(H5) outbreaks detected in 24 provinces in Vietnam from 2017–2022.

No.	Provinces	Region	Active live bird market surveillance					Passive outbreak surveillance	
			Years sampled	No. of sampling rounds	No. of live bird markets	Mean (SD) no. of pooled^a^ samples per month	Total no. of pooled^a^ samples	Years when an outbreak was detected	Total no. of A(H5) outbreaks^b^
1	Bac Giang	North	2022	9	4	28.0 (0.0)	252	–	0
2	Bac Ninh	North	2017–2022	15	9	32.8 (6.1)	492	2020–2021	24
3	Cao Bang	North	2019–2022	45	11	28.7 (7.6)	1176	2017–2018, 2021	6
4	Ha Giang	North	2022	7	4	27.9 (0.4)	195	2021	2
5	Ha Noi	North	2017–2022	60	11	31.8 (10.5)	1815	2020–2022	27
6	Hai Phong	North	2017–2022	61	8	30.7 (6.7)	1809	2018, 2020	5
7	Lang Son	North	2017–2022	61	14	63.2 (25.0)	3728	2021	18
8	Lao Cai	North	2018–2022	55	6	32.1 (9.6)	1575	2022	1
9	Nam Dinh	North	2017–2022	51	9	29.5 (7.0)	1416	2020–2022	8
10	Ninh Binh	North	2022	9	5	28.0 (0.0)	252	2020–2022	26
11	Quang Ninh	North	2017–2022	61	8	31.7 (7.6)	1809	2017–2018, 2020–2021	20
12	Thai Binh	North	2022	7	4	28.0 (0.0)	196	2019–2022	7
13	Thai Nguyen	North	2022	9	4	28.0 (0.0)	252	2021	2
14	Dak Lak	Central	2018–2022	55	7	30.9 (7.7)	1575	2017–2018, 2021	12
15	Nghe An	Central	2018–2022	55	10	31.5 (9.1)	1575	2018, 2020–2021	24
16	Quang Nam	Central	2017–2022	61	6	31.8 (8.3)	1812	2019, 2021–2022	11
17	Quang Ngai	Central	2022	7	4	28.0 (0.0)	196	2020–2022	7
18	Quang Tri	Central	2017	6	5	40.0 (0.0)	240	2017, 2021–2022	9
19	Thanh Hoa	Central	2022	7	4	32.7 (21.1)	196	2020	17
20	Thua Thien Hue	Central	2017	6	5	40.0 (10.1)	240	–	0
21	Can Tho	South	2019–2022	45	6	29.4 (11.5)	1176	2017, 2020	3
22	Dong Thap	South	2022	9	5	28.0 (0.0)	252	2020–2021	5
23	Tien Giang	South	2022	57	7	32.6 (11.4)	1730	2020–2022	9
24	Vinh Long	South	2017–2022	61	11	31.3 (8.2)	1815	2017, 2020	3
Total				819	167	32.4 (7.3)	25,774		246

Abbreviation: SD, standard deviation.

^a^ One pooled sample contained five single samples collected from the same poultry seller (samples of this poultry seller will not be pooled with samples from other poultry sellers) at the same market during the same visit. Each single swab is from a different animal or different environmental sample. Samples are pooled in the field and then transported to the laboratory.

^b^ A(H5) outbreaks were of influenza A(H5N1), A(H5N6), or A(H5N8) in poultry.

**FIGURE 1:**
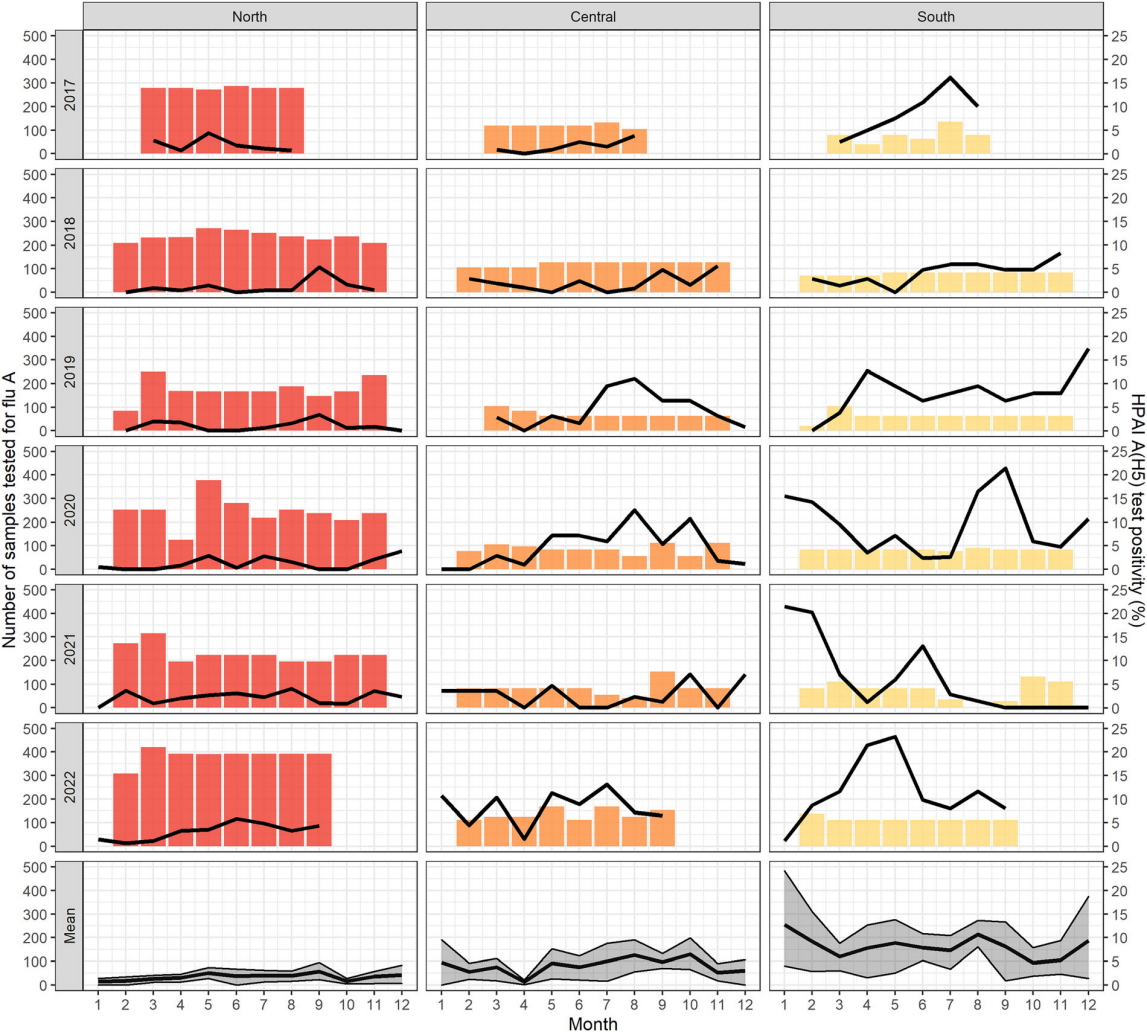
Temporal distribution of influenza A(H5) viruses detected in live bird markets within each region. The numbers of samples that were tested in each province each month for influenza A and used as the denominator for A(H5) monthly percent positivity are indicated by the colored bars. The colors of the bars represent the overall region of Vietnam where red indicates the Northern region, orange the Central region, and yellow the Southern region. Monthly influenza A(H5) percent positivity within each region and year is indicated by the black lines. The bottom row indicates the mean monthly A(H5) percent positivity and corresponding 95% confidence interval within each region, averaging across all 6 years.

**FIGURE 2:**
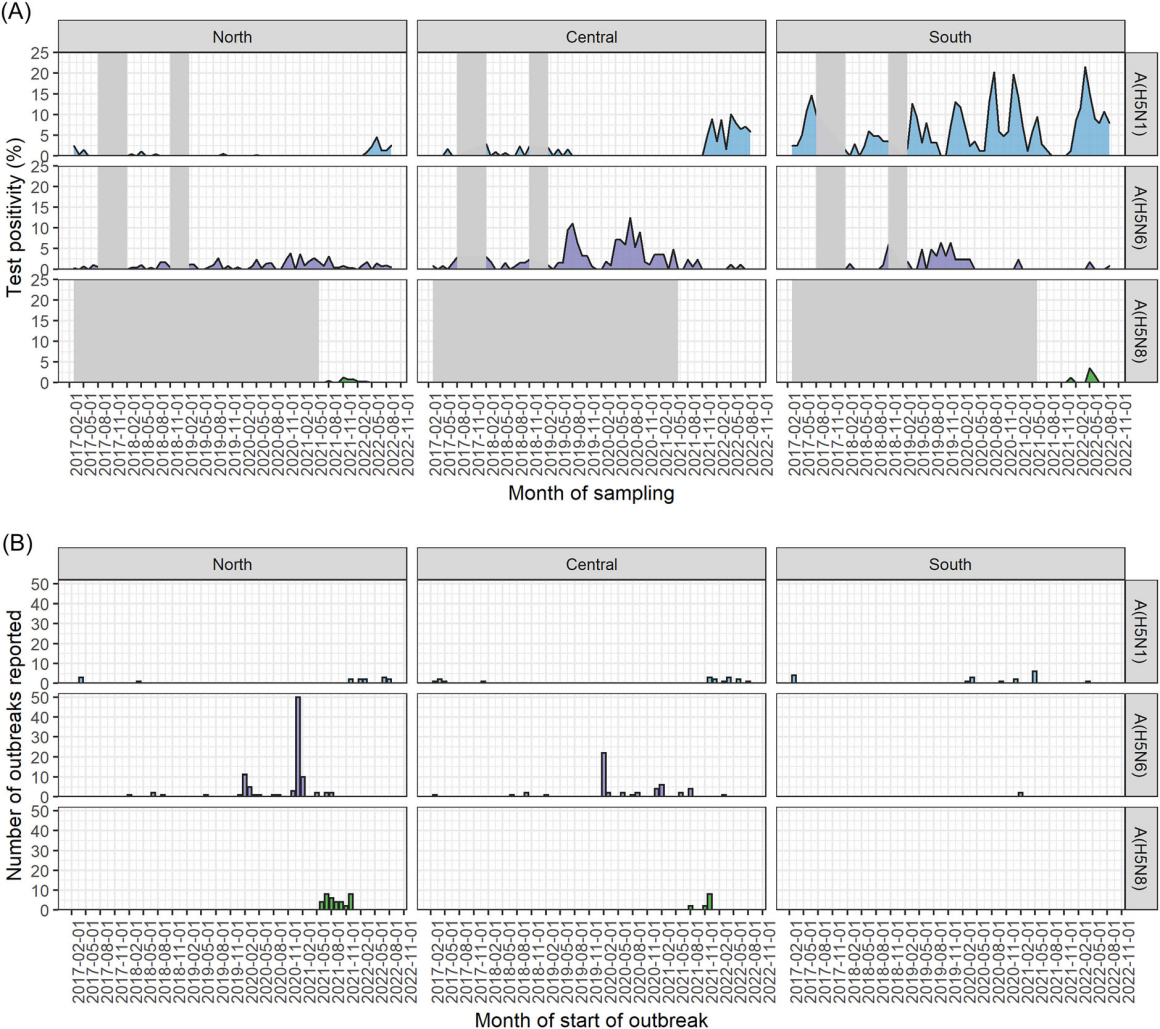
Temporal distribution of influenza A(H5N1), A(H5N6), and A(H5N8) viruses detected in three regions of Vietnam. (A) Monthly percent positivity of influenza samples that were confirmed as positive for influenza A(H5N1), A(H5N6), or A(H5N8) by rRT‐PCR and collected through active surveillance for avian influenza in live bird markets. Shaded areas indicate lack of surveillance activity. (B) Outbreaks of influenza A(H5N1), A(H5N6), and A(H5N8) reported to the Vietnam Department of Animal Health each month.

Overall, 9522 (36.9%, 95% CI: 36.4–37.5) pooled samples were positive for influenza A and 938 (3.6%, 95% CI: 3.4–3.9) were positive for A(H5) virus (Table 2). Influenza A(H5) positivity differed over time and by region, with the Southern region having the highest positivity (Figures 1 and S1). In total, 500 (1.9%; 95% CI: 1.8–2.1) pooled samples from poultry and their environment were infected with influenza A(H5N1), 287 (1.1%; 95% CI: 1.0–1.2) with A(H5N6), and 19 (0.2%; 95% CI: 0.1–0.4) with A(H5N8) (Table 3). A total of 23 pooled samples had both A(H5N1) and A(H5N6) viruses detected, three A(H5N1) and A(H5N8) viruses detected, and one A(H5N6) and A(H5N8) viruses detected. In the Southern region of Vietnam, influenza A(H5N1) percent positivity increased over time while influenza A(H5N6) increased from late 2018 to mid‐2020, with only sporadic detection in subsequent years (Figure 2). Influenza A(H5N8) virus was first detected in Vietnam in June 2021 but has not been detected in LBMs since July 2022 (Figure 2). No definitive patterns of A(H5), A(H5N1), A(H5N6), and A(H5N8) virus seasonality were observed (Figures 1 and 2 and Tables 2 and 3).

**TABLE 2:** Influenza A and A(H5) percent positivity of pooled samples identified in active surveillance in live bird markets, stratified by surveillance period, host species, sample type, and market type.

Level	Flu A			A(H5)	
	No. tested	No. positive	% Positive (95% CI)	No. positive	% Positive (95% CI)
Year					
2017	2877	844	29.3 (27.7, 31.0)	90	3.1 (2.6, 3.8)
2018	4370	1475	33.8 (32.4, 35.2)	85	1.9 (1.6, 2.4)
2019	3528	1390	39.4 (37.8, 41.0)	119	3.4 (2.8, 4.0)
2020	4809	1876	39.0 (37.6, 40.4)	175	3.6 (3.1, 4.2)
2021	4620	1751	37.9 (36.5, 39.3)	144	3.1 (2.7, 3.7)
2022	5570	2186	39.2 (38.0, 40.5)	325	5.8 (5.2, 6.5)
Month					
Jan	1092	353	32.3 (29.6, 35.2)	41	3.8 (2.8, 5.1)
Feb	1902	658	34.6 (32.5, 36.8)	66	3.5 (2.7, 4.4)
Mar	2960	1003	33.9 (32.2, 35.6)	84	2.8 (2.3, 3.5)
Apr	2467	867	35.1 (33.3, 37.0)	68	2.8 (2.2, 3.5)
May	2856	1084	38.0 (36.2, 39.7)	128	4.5 (3.8, 5.3)
June	2698	1029	38.1 (36.3, 40.0)	98	3.6 (3.0, 4.4)
Jul	2670	1024	38.4 (36.5, 40.2)	114	4.3 (3.6, 5.1)
Aug	2494	993	39.8 (37.9, 41.8)	107	4.3 (3.6, 5.2)
Sep	2177	898	41.2 (39.2, 43.3)	99	4.5 (3.7, 5.5)
Oct	1533	560	36.5 (34.2, 39.0)	39	2.5 (1.9, 3.5)
Nov	1637	560	34.2 (32.0, 36.5)	43	2.6 (2.0, 3.5)
Dec	1288	493	38.3 (35.7, 41.0)	51	4.0 (3.0, 5.2)
Species					
Chicken	14,888	5419	36.4 (35.6, 37.2)	267	1.8 (1.6, 2.0)
Duck	6407	2642	41.2 (40.0, 42.4)	465	7.3 (6.6, 7.9)
Muscovy duck	439	163	37.1 (32.7, 41.7)	60	13.7 (10.8, 17.2)
Poultry (mixed)	142	61	43.0 (35.1, 51.2)	7	4.9 (2.4, 9.8)
Environment	3898	1237	31.7 (30.3, 33.2)	139	3.6 (3.0, 4.2)
Type of sample					
Poultry swab	21,876	8285	37.9 (37.2, 38.5)	799	3.7 (3.4, 3.9)
Poultry feces	3643	1180	32.4 (30.9, 33.9)	135	3.7 (3.1, 4.4)
Water	255	57	22.4 (17.7, 27.9)	4	1.6 (0.6, 4.0)
Type of market					
Traditional	21,298	7854	36.9 (36.2, 37.5)	798	3.7 (3.5, 4.0)
Non‐traditional	4476	1668	37.3 (35.9, 38.7)	140	3.1 (2.7, 3.7)
Total	25,774	9522	36.9 (36.4, 37.5)	938	3.6 (3.4, 3.9)

**TABLE 3:** Influenza A(H5N1), A(H5N6), and A(H5N8) percent positivity of pooled samples identified in active surveillance in live bird markets, stratified by surveillance period, host species, sample type, and market type.

Level	No. tested	A(H5N1)		A(H5N6)		A(H5N8)	
		No. positive	% Positive (95% CI)	No. positive	% Positive (95% CI)	No. positive	% Positive^a^ (95% CI)
Year							
2017	2877	56	1.9 (1.5, 2.5)	15	0.5 (0.3, 0.9)	–	–
2018	4370	40	0.9 (0.7, 1.2)	37	0.8 (0.6, 1.2)	–	–
2019	3528	41	1.2 (0.9, 1.6)	63	1.8 (1.4, 2.3)	–	–
2020	4809	78	1.6 (1.3, 2.0)	79	1.6 (1.3, 2.0)	–	–
2021	4620	50	1.1 (0.8, 1.4)	59	1.3 (1.0, 1.6)	6	0.2 (0.1, 0.5)
2022	5570	235	4.2 (3.7, 4.8)	34	0.6 (0.4, 0.9)	13	0.2 (0.1, 0.4)
Month							
Jan	1092	28	2.6 (1.8, 3.7)	7	0.6 (0.3, 1.3)	4	0.8 (0.3, 2.1)
Feb	1902	42	2.2 (1.6, 3.0)	20	1.1 (0.7, 1.6)	1	0.2 (0.0, 1.0)
Mar	2960	52	1.8 (1.3, 2.3)	26	0.9 (0.6, 1.3)	1	0.2 (0.0, 0.9)
Apr	2467	46	1.9 (1.4, 2.5)	18	0.7 (0.5, 1.2)	1	0.2 (0.0, 0.9)
May	2856	70	2.5 (1.9, 3.1)	36	1.3 (0.9, 1.7)	4	0.6 (0.2, 1.5)
June	2698	60	2.2 (1.7, 2.9)	23	0.9 (0.6, 1.3)	2	0.2 (0.1, 0.7)
Jul	2670	58	2.2 (1.7, 2.8)	28	1.0 (0.7, 1.5)	0	0.0 (0.0, 0.4)
Aug	2494	53	2.1 (1.6, 2.9)	40	1.6 (1.2, 2.2)	1	0.1 (0.0, 0.6)
Sep	2177	54	2.5 (1.9, 3.2)	27	1.2 (0.9, 1.8)	0	0.0 (0.0, 0.4)
Oct	1533	9	0.6 (0.3, 1.1)	21	1.4 (0.9, 2.1)	0	0.0 (0.0, 0.9)
Nov	1637	10	0.6 (0.3, 1.1)	24	1.5 (1.0, 2.2)	3	0.7 (0.2, 2.1)
Dec	1288	18	1.4 (0.9, 2.2)	17	1.3 (0.8, 2.1)	2	0.4 (0.1, 1.6)
Species							
Chicken	14,888	121	0.8 (0.7, 1.0)	82	0.6 (0.4, 0.7)	6	0.1 (0.1, 0.3)
Duck	6407	261	4.1 (3.6, 4.6)	150	2.3 (2.0, 2.7)	10	0.5 (0.3, 0.9)
Muscovy duck	439	47	10.7 (8.1, 13.9)	14	3.2 (1.9, 5.3)	1	1.1 (0.2, 5.7)
Poultry (mixed)	142	1	0.7 (0.1, 3.9)	6	4.2 (2.0, 8.9)	0	0.0 (0.0, 43.4)
Environment	3898	70	1.8 (1.4, 2.3)	35	0.9 (0.6, 1.2)	2	0.2 (0.0, 0.6)
Type of sample							
Poultry swab	21,876	430	2.0 (1.8, 2.2)	252	1.2 (1.0, 1.3)	17	0.2 (0.2, 0.4)
Poultry feces	3643	70	1.9 (1.5, 2.4)	34	0.9 (0.7, 1.3)	2	0.2 (0.0, 0.6)
Water^b^	255	0	0.0 (0.0, 1.5)	1	0.4 (0.1, 2.2)	–	–
Type of market							
Traditional	21,298	385	1.8 (1.6, 2.0)	269	1.3 (1.1, 1.4)	19	0.3 (0.2, 0.4)
Non‐traditional	4476	115	2.6 (2.1, 3.1)	18	0.4 (0.3, 0.6)	0	0.0 (0.0, 0.3)
Total	25,774	500	1.9 (1.8, 2.1)	287	1.1 (1.0, 1.2)	19	0.2 (0.1, 0.4)

^a^ A(H5N8) was only present in Vietnam from June 2021 onward, so only data from June 2021–September 2022 (*n* = 8202 observations) are included in the denominator used to calculate the percent positive.

^b^ Samples were only collected from water in 2017, which was not when A(H5N8) was circulating in Vietnam.

During this same period, 246 A(H5) virus outbreaks were reported in poultry through passive surveillance (Table 1); 146 (59.3%) A(H5) virus outbreaks occurred in Northern Vietnam, 80 (32.5%) occurred in the Central region, and 20 (8.1%) occurred in the Southern region. A total of 50 (20.3%) reported outbreaks were of influenza A(H5N1), 148 (60.2%) A(H5N6), and 48 (19.5%) A(H5N8) viruses. The number of influenza A(H5N1), A(H5N6), and A(H5N8) virus outbreaks reported was not statistically correlated temporally with the percent of samples positive for influenza A(H5N1), A(H5N6), and A(H5N8) in active LBM surveillance (A(H5N1) *ρ* = 0.4, A(H5N6) *ρ* = 0.4, and A(H5N8) *ρ* = 0.3); results differed some in the sensitivity analysis with a 30‐day time lag for detections in LBMs (A(H5N1) *ρ* = 0.04, A(H5N6) *ρ* = 0.5, and A(H5N8) *ρ* = 0.4). While there were some regional overlaps between increased periods of high A(H5) virus activity in LBMs and outbreaks detected (e.g., A(H5N6) in Northern regions during November 2020), these trends were not correlated over the study period (Figure 2).

The total number of samples positive for influenza A(H5), A(H5N1), A(H5N6), and A(H5N8) virus in LBMs varied by time and provincial level. More pooled poultry and environmental samples were tested (*n* = 5570 pooled samples) and had a higher percent positivity for A(H5) virus (5.8%, 95% CI: 5.2–6.5) during 2022 compared with any other year (Table 2). In 2022, at the province level, up to 28.6% (95% CI: 23.3–34.4) of pooled poultry and environmental samples tested positive for A(H5) virus (Table S2). This increase in A(H5) percent positivity in 2022 was largely associated with an increase in A(H5N1) viruses with 4.2% (95% CI: 3.7% to 4.8%) of poultry samples testing positive for influenza A(H5N1) virus that year (Table 3). Due to heightened concern about increasing global outbreaks of A(H5) virus in poultry, new provinces were added to the active surveillance for avian influenza program in 2022; however, the number of positive pooled poultry samples did not correlate with monthly influenza A(H5) virus percent positivity at the provincial or regional levels (Figures 1 and S2).

Influenza A(H5N1), A(H5N6), and A(H5N8) virus percent positivity was highest in samples collected from Muscovy ducks (Table 3). Influenza A(H5N1), A(H5N6), and A(H5N8) viruses were detected in both poultry and environmental samples, although there were differences in virus percent positivity across poultry species. Influenza A(H5N1) virus was found more often in pooled poultry samples from non‐traditional LBMs, whereas A(H5N6) and A(H5N8) viruses were more commonly detected in traditional LBMs. No samples from non‐traditional markets were positive for influenza A(H5N8) (Table 3).

## 4 DISCUSSION

Using monthly sampling of poultry in LBMs in Northern, Central, and Southern Vietnam, we assessed trends in influenza A, A(H5), A(H5N1), A(H5N6), and A(H5N8) percent positivity over nearly 6 years. Active surveillance detected A(H5) viruses in LBMs throughout the year and in most sampled provinces. Influenza A(H5) and A(H5N1) percent positivity increased in 2022, aligning with the recent global increase in A(H5N1) detections in poultry and wild birds. Many of the A(H5N1) detections in LBMs occurred in ducks in Southern Vietnam. Influenza A(H5) viruses were actively detected more often in LBMs than were passively reported following outbreaks. Although there were no statistically significant correlations between high positivity in LBMs and increases in outbreaks in each region or time period, possibly due to biases in outbreak detection and reporting and quick culling post‐detection of outbreaks, further molecular characterization of viruses detected in each setting may highlight epidemiologic links between farms and markets. These data will be important to assess transmission dynamics and potential mitigations to slow the spread of avian influenza viruses and reduce disease burden.

We saw an increase in A(H5) percent positivity in 2022, with over 6% of pooled poultry and environmental samples testing positive for the virus that year. Most of those positive samples were attributable to A(H5N1) viruses. Starting in 2021, there has been a global increase in influenza A(H5N1) outbreaks in birds, predominantly of clade 2.3.4.4b.^16^ This increase in poultry outbreaks and wild bird detections has led to many exposures in people handling infected birds, and from January 2022 to June 2023, human cases of A(H5N1) have been reported from seven countries.^16^ While there has been a low number of human A(H5N1) cases detected amidst the large number of poultry outbreaks,^17^ some cases have led to severe illness.^16^, ^18^

Influenza A(H5) viruses continue to pose a significant health threat to the public and economic burden to the poultry sector.^9^, ^19^ Results from this study provide information on influenza viruses that have been circulating in LBMs. While avian influenza A(H3), A(H5), A(H6), A(H7), A(H9), and A(H10) viruses have infected individuals, the majority of human infections with avian influenza viruses have been due to influenza A(H5N1) and A(H7N9).^20^ Since 1997, nearly 900 human A(H5N1) cases have been identified with a cumulative case fatality proportion of over 50%.^20^, ^21^ Most human cases of A(H5N1) infection have been detected in individuals who have come in direct contact with sick or dead birds in areas with a high concentration of infectious virus, like LBMs.^16^ These settings host a wide diversity of avian influenza subtypes and clades, increasing the risk of viral reassortment and the potential for human infection.^22^ Thus, active surveillance for avian influenza in LBMs remains important for situational awareness in areas with high potential for animal‐to‐human spillover of avian influenza viruses.

Influenza A(H5) detection by active surveillance varied by species, highlighting areas to intervene to reduce avian influenza in birds and exposure to humans. Across sampled poultry species in LBMs, Muscovy ducks had the highest A(H5) positivity rate of 14%, followed by general ducks (7%) and chickens (2%); this trend across species was similar for influenza A(H5N1), A(H5N6), and A(H5N8) viruses. Our finding aligns with prior active surveillance in Vietnamese LBMs that found higher A(H5N1) prevalence in ducks compared with chickens.^9^, ^19^ Furthermore, influenza A(H5) has been observed to circulate asymptomatically in poultry at LBMs and farms,^23^ especially among ducks.^9^, ^19^, ^23^ Because of this, active surveillance is important for capturing asymptomatic cases of A(H5) in the duck species.

We found differences in geographic and annual temporal patterns of avian influenza. We observed no seasonal trends in when A(H5) was detected in LBMs but the prevalence of viral subtypes varied over time. Our results are consistent with previous publications that found year‐round circulation of A(H5) virus in Vietnam.^9^, ^19^ Influenza A(H5N1) and A(H5N6) viruses circulated across all sampled regions, with a higher A(H5N1) positivity rate observed in the Southern region and a higher A(H5N6) positivity rate in the Central region. Influenza A(H5N8) virus was only detected in the Northern and Southern areas. The A(H5) positivity rate was relatively higher in the Southern provinces reflecting the higher number of domestic ducks living in these areas alongside a high density of both domestic and wild waterfowl birds.

This study had limitations. Avian influenza sampling was conducted during different month ranges and provinces each year which limited our ability to use modeling techniques to assess temporal patterns in avian influenza. However, we found no consistent seasonality pattern in provinces with consistent monthly sampling (Figure S2). Each month, convenience sampling was conducted on a sample of poultry from separate vendors in LBMs, so it is possible that some A(H5) virus detections were missed. We were only able to include A(H5) outbreaks reported to the Vietnamese DAH, and some outbreaks may not have been reported. However, we believe underreporting of outbreaks was minimal as outbreaks are considered a reportable event. Influenza clade results were not available at the time of this analysis but will be included in future analyses of this surveillance data.

Avian influenza has been a growing global concern for both the poultry industry, wild bird populations, and human health. We found that influenza A(H5) virus was detected in LBMs at least once in most provinces sampled in Vietnam from 2017 to 2022, with these detections capturing more A(H5) virus activity than what was passively reported through outbreaks. Because LBMs are an area of high concern for avian influenza spillover events to humans, it is important for active surveillance activities to continue to supplement avian influenza detections from outbreaks.

## AUTHOR CONTRIBUTIONS

Diep T. Nguyen, Kelsey M. Sumner, Thoa T. M. Nguyen, Sonja J. Olsen, Philip L. Gould, Long V. Nguyen, and Charles Todd Davis conceptualized the manuscript. Diep T. Nguyen, Kelsey M. Sumner, Thoa T. M. Nguyen, and Philip L. Gould provided visualization and data analysis. Tien M. Hoang, Chuong D. Vo, Tho D. Nguyen, Phuong T. Nguyen, Genyan Yang, Yunho Jang, and Joyce Jones provided data management, data curation, and/or laboratory analysis. Thoa T. M. Nguyen provided project administration. Minh Q. Phan, Sonja J. Olsen, Philip L. Gould, Long V. Nguyen, and Charles Todd Davis provided supervision. Diep T. Nguyen, Kelsey M. Sumner, and Thoa T. M. Nguyen wrote the original draft of the manuscript. Minh Q. Phan, Tien M. Hoang, Chuong D. Vo, Tho D. Nguyen, Phuong T. Nguyen, Genyan Yang, Yunho Jang, Joyce Jones, Sonja J. Olsen, Philip L. Gould, Long V. Nguyen, and Charles Todd Davis reviewed and edited the manuscript.

## CONFLICT OF INTEREST STATEMENT

All authors report no conflicts of interest to declare.

## ETHICS STATEMENT

All animal sampling was conducted under the guidance and approval of the CDC's Institutional Animal Care and Use Committee and Animal Care and Use Program Office.

### PEER REVIEW

The peer review history for this article is available at https://www.webofscience.com/api/gateway/wos/peer-review/10.1111/irv.13245.

## DISCLAIMER

The findings and conclusions in this report are those of the authors and do not necessarily represent the views of CDC. Some authors are federal employees of the United States government, and this work was prepared as part of their official duties. Title 17 U.S.C. 105 provides that “copyright protection under this title is not available for any work of the United States Government.”

## Supporting information


**Table S1.** Prevalence of influenza A, A(H5), A(H5N1), A(H5N6), and A(H5N8) viruses by year at the live bird market, commune, district, and provincial levels.
**Table S2.** Influenza A(H5) test positivity in 2022 across provinces included in active surveillance in live bird markets.
**Figure S1.** Spatial distribution of influenza A(H5N1), A(H5N6), and A(H5N8) viruses detected in live bird markets. Provinces where surveillance was not conducted are shaded grey. Surveillance of A(H5N8) was not conducted from 2017–2020.
**Figure S2.** Influenza A(H5) sampling and test positivity from 2017–2022 across provinces that participated in active surveillance in live bird markets. The number of samples each province tested for influenza A(H5) each month is indicated by the colored bars. The colors of the bars represent the region of Vietnam of each province where red indicates the North, orange Central, and yellow South. Monthly influenza A(H5) test positivity within each province is indicated by the black lines.

## Data Availability

A summarized version of the data may be available upon request.
