# Supplementary material for: Avian influenza A(H5) virus circulation in live bird markets in Vietnam, 2017–2022
Source: Influenza Other Respir Viruses. 2023 Dec 27;17(12):e13245. doi: 10.1111/irv.13245 (PMC10752245; doi:10.1111/irv.13245)

**SUPPLEMENTAL MATERIAL**

**Supplemental Table 1.** **Prevalence of influenza A, A(H5), A(H5N1), A(H5N6), and A(H5N8) viruses by year at the live bird market, commune, district, and provincial levels**

| **Level** | **Total No.** | **Flu A** | |  | **A(H5)** | |  | **A(H5N1)** | |  | **A(H5N6)** | |  | **A(H5N8)** | |
| --- | --- | --- | --- | --- | --- | --- | --- | --- | --- | --- | --- | --- | --- | --- | --- |
|  |  | **No. with ≥1 positive result** | **% with ≥1 positive result**  **(95% CI)** |  | **No. with ≥1 positive result** | **% with ≥1 positive result**  **(95% CI)** |  | **No. with ≥1 positive result** | **% with ≥1 positive result**  **(95% CI)** |  | **No. with ≥1 positive result** | **% with ≥1 positive result**  **(95% CI)** |  | **No. with ≥1 positive result** | **% with ≥1 positive result**  **(95% CI)** |
| **2017** | |  |  |  |  |  |  |  |  |  |  |  |  |  |  |
| Province | 11 | 11 | 100.0 (74.1, 100.0) |  | 10 | 90.9 (62.3, 98.4) |  | 8 | 72.7 (43.4, 90.3) |  | 5 | 45.5 (21.3, 72.0) |  | – | – |
| District | 43 | 43 | 100.0 (91.8, 100.0) |  | 19 | 44.2 (30.4, 58.9) |  | 14 | 32.6 (20.5, 47.5) |  | 7 | 16.3 (8.1, 30.0) |  | – | – |
| Commune | 55 | 55 | 100.0 (93.5, 100.0) |  | 22 | 40.0 (28.1, 53.2) |  | 15 | 27.3 (17.3, 40.2) |  | 7 | 12.7 (6.3, 24.0) |  | – | – |
| Market | 54 | 54 | 100.0 (93.4, 100.0) |  | 20 | 37.0 (25.4, 50.4) |  | 15 | 27.8 (17.6, 40.9) |  | 7 | 13.0 (6.4, 24.4) |  | – | – |
| **2018** | |  |  |  |  |  |  |  |  |  |  |  |  |  |  |
| Province | 10 | 10 | 100.0 (72.2, 100.0) |  | 8 | 80.0 (49.0, 94.3) |  | 6 | 60.0 (31.3, 83.2) |  | 8 | 80.0 (49.0, 94.3) |  | – | – |
| District | 49 | 48 | 98.0 (89.3, 99.6) |  | 27 | 55.1 (41.3, 68.1) |  | 15 | 30.6 (19.5, 44.5) |  | 18 | 36.7 (24.7, 50.7) |  | – | – |
| Commune | 63 | 60 | 95.2 (86.9, 98.4) |  | 30 | 47.6 (35.8, 59.7) |  | 15 | 23.8 (15.0, 35.6) |  | 21 | 33.3 (22.9, 45.6) |  | – | – |
| Market | 66 | 62 | 93.9 (85.4, 97.6) |  | 30 | 45.5 (34.0, 57.4) |  | 15 | 22.7 (14.3, 34.2) |  | 21 | 31.8 (21.8, 43.8) |  | – | – |
| **2019** | |  |  |  |  |  |  |  |  |  |  |  |  |  |  |
| Province | 13 | 13 | 100.0 (77.2, 100.0) |  | 10 | 76.9 (49.7, 91.8) |  | 5 | 38.5 (17.7, 64.5) |  | 10 | 76.9 (49.7, 91.8) |  | – | – |
| District | 38 | 38 | 100.0 (90.8, 100.0) |  | 21 | 55.3 (39.7, 69.9) |  | 7 | 18.4 (9.2, 33.4) |  | 18 | 47.4 (32.5, 62.7) |  | – | – |
| Commune | 41 | 41 | 100.0 (91.4, 100.0) |  | 21 | 51.2 (36.5, 65.7) |  | 7 | 17.1 (8.5, 31.3) |  | 18 | 43.9 (29.9, 59.0) |  | – | – |
| Market | 42 | 42 | 100.0 (91.6, 100.0) |  | 22 | 52.4 (37.7, 66.6) |  | 7 | 16.7 (8.3, 30.6) |  | 18 | 42.9 (29.1, 57.8) |  | – | – |
| **2020** | |  |  |  |  |  |  |  |  |  |  |  |  |  |  |
| Province | 13 | 13 | 100.0 (77.2, 100.0) |  | 11 | 84.6 (57.8, 95.7) |  | 4 | 30.8 (12.7, 57.6) |  | 8 | 61.5 (35.5, 82.3) |  | – | – |
| District | 48 | 48 | 100.0 (92.6, 100.0) |  | 32 | 66.7 (52.5, 78.3) |  | 10 | 20.8 (11.7, 34.3) |  | 22 | 45.8 (32.6, 59.7) |  | – | – |
| Commune | 57 | 55 | 96.5 (88.1, 99.0) |  | 33 | 57.9 (45.0, 69.8) |  | 11 | 19.3 (11.1, 31.3) |  | 22 | 38.6 (27.1, 51.6) |  | – | – |
| Market | 59 | 57 | 96.6 (88.5, 99.1) |  | 33 | 55.9 (43.3, 67.8) |  | 11 | 18.6 (10.7, 30.4) |  | 22 | 37.3 (26.1, 50.0) |  | – | – |
| **2021** | |  |  |  |  |  |  |  |  |  |  |  |  |  |  |
| Province | 13 | 13 | 100.0 (77.2, 100.0) |  | 12 | 92.3 (66.7, 98.6) |  | 4 | 30.8 (12.7, 57.6) |  | 11 | 84.6 (57.8, 95.7) |  | 2 | 15.4 (4.3, 42.2) |
| District | 48 | 46 | 95.8 (86.0, 98.8) |  | 30 | 62.5 (48.4, 74.8) |  | 9 | 18.8 (10.2, 31.9) |  | 20 | 41.7 (28.8, 55.7) |  | 4 | 8.3 (3.3, 19.6) |
| Commune | 60 | 56 | 93.3 (84.1, 97.4) |  | 33 | 55.0 (42.5, 66.9) |  | 10 | 16.7 (9.3, 28.0) |  | 21 | 35.0 (24.2, 47.6) |  | 4 | 6.7 (2.6, 15.9) |
| Market | 59 | 56 | 94.9 (86.1, 98.3) |  | 33 | 55.9 (43.3, 67.8) |  | 10 | 16.9 (9.5, 28.5) |  | 21 | 35.6 (24.6, 48.3) |  | 4 | 6.8 (2.7, 16.2) |
| **2022** | |  |  |  |  |  |  |  |  |  |  |  |  |  |  |
| Province | 22 | 22 | 100.0 (85.1, 100.0) |  | 18 | 81.8 (61.5, 92.7) |  | 17 | 77.3 (56.6, 89.9) |  | 9 | 30.9 (23.3, 61.3) |  | 4 | 18.2 (7.3, 38.5) |
| District | 81 | 78 | 96.3 (89.7, 98.7) |  | 52 | 64.2 (53.3, 73.8) |  | 44 | 54.3 (43.5, 64.7) |  | 19 | 23.5 (15.6, 33.8) |  | 4 | 4.9 (1.9, 12.0) |
| Commune | 93 | 90 | 96.8 (90.9, 98.9) |  | 58 | 62.4 (52.2, 71.5) |  | 48 | 51.6 (41.6, 61.5) |  | 20 | 21.5 (14.4, 30.9) |  | 5 | 5.4 (2.3, 12.0) |
| Market | 101 | 96 | 95.0 (88.9, 97.9) |  | 59 | 58.4 (48.7, 67.5) |  | 50 | 49.5 (40.0, 59.1) |  | 20 | 19.8 (13.2, 28.6) |  | 5 | 5.0 (2.1, 11.1) |
| **Total period 2017 to 2022** | | | |  |  |  |  |  |  |  |  |  |  |  |  |
| Province | 24 | 24 | 100.0 (86.2, 100.0) |  | 22 | 91.7 (74.2, 97.7) |  | 19 | 79.2 (59.5, 90.8) |  | 19 | 79.2 (59.5, 90.8) |  | 5 | 20.8 (9.2, 40.5) |
| District | 112 | 111 | 99.1 (95.1, 99.8) |  | 72 | 64.3 (55.1, 72.6) |  | 56 | 50.0 (40.9, 59.1) |  | 53 | 47.3 (38.3, 56.5) |  | 7 | 6.2 (3.1, 12.3) |
| Commune | 153 | 150 | 98.0 (94.4, 99.3) |  | 90 | 58.8 (50.9, 66.3) |  | 67 | 43.8 (36.2, 51.7) |  | 59 | 38.6 (31.2, 46.5) |  | 8 | 5.2 (2.7, 10.0) |
| Market | 164 | 159 | 97.0 (93.1, 98.7) |  | 90 | 54.9 (47.2, 62.3) |  | 67 | 40.9 (33.6, 48.5) |  | 59 | 36.0 (29.0, 43.6) |  | 8 | 4.9 (2.5, 9.3) |

Abbreviations: CI, Confidence interval

**Supplemental Table 2. Influenza A(H5) test positivity in 2022 across provinces included in active surveillance in live bird markets**

| **No.** | **Provinces** | **No. tested** | **No. positive** | **% Positive (95% CI)** |
| --- | --- | --- | --- | --- |
| 1 | Bac Giang^a^ | 252 | 0 | 0 (0.0, 1.5) |
| 2 | Bac Ninh | 252 | 16 | 6.3 (3.9, 10.1) |
| 3 | Can Tho | 252 | 14 | 5.6 (3.3, 9.1) |
| 4 | Cao Bang | 252 | 21 | 8.3 (5.5, 12.4) |
| 5 | Dak Lak | 252 | 27 | 10.7 (7.5, 15.1) |
| 6 | Dong Thap^a^ | 252 | 17 | 6.7 (4.3, 10.5) |
| 7 | Ha Giang^a^ | 195 | 11 | 5.6 (3.2, 9.8) |
| 8 | Ha Noi | 252 | 0 | 0.0 (0.0, 1.5) |
| 9 | Hai Phong | 252 | 20 | 7.9 (5.2, 11.9) |
| 10 | Lang Son | 504 | 13 | 2.6 (1.5, 4.4) |
| 11 | Lao Cai | 252 | 15 | 6.0 (3.6, 9.6) |
| 12 | Nam Dinh | 252 | 6 | 2.4 (1.1, 5.1) |
| 13 | Nghe An | 252 | 2 | 0.8 (0.2, 2.8) |
| 14 | Ninh Binh^a^ | 252 | 3 | 1.2 (0.4, 3.4) |
| 15 | Quang Nam | 252 | 12 | 4.8 (2.7, 8.1) |
| 16 | Quang Ngai^a^ | 196 | 45 | 23.0 (17.6, 29.3) |
| 17 | Quang Ninh | 252 | 0 | 0.0 (0.0, 1.5) |
| 18 | Thai Binh^a^ | 196 | 6 | 3.1 (1.4, 6.5) |
| 19 | Thai Nguyen^a^ | 252 | 0 | 0.0 (0.0, 1.5) |
| 20 | Thanh Hoa^a^ | 196 | 10 | 5.1 (2.8, 9.1) |
| 21 | Tien Giang | 251 | 15 | 6.0 (3.7, 9.6) |
| 22 | Vinh Long | 252 | 72 | 28.6 (23.3, 34.4) |

^a^2022 was the first year this province was included in live bird market surveillance. Note that influenza A(H5) test positivity was 6.2% for provinces that were included in live bird market surveillance prior to 2022 and 5.7% for provinces newly included in surveillance starting in 2022.

**Supplemental Figure 1.** **Spatial distribution of influenza A(H5N1), A(H5N6), and A(H5N8) viruses detected in live bird markets.** Provinces where surveillance was not conducted are shaded grey. Surveillance of A(H5N8) was not conducted from 2017–2020.

**
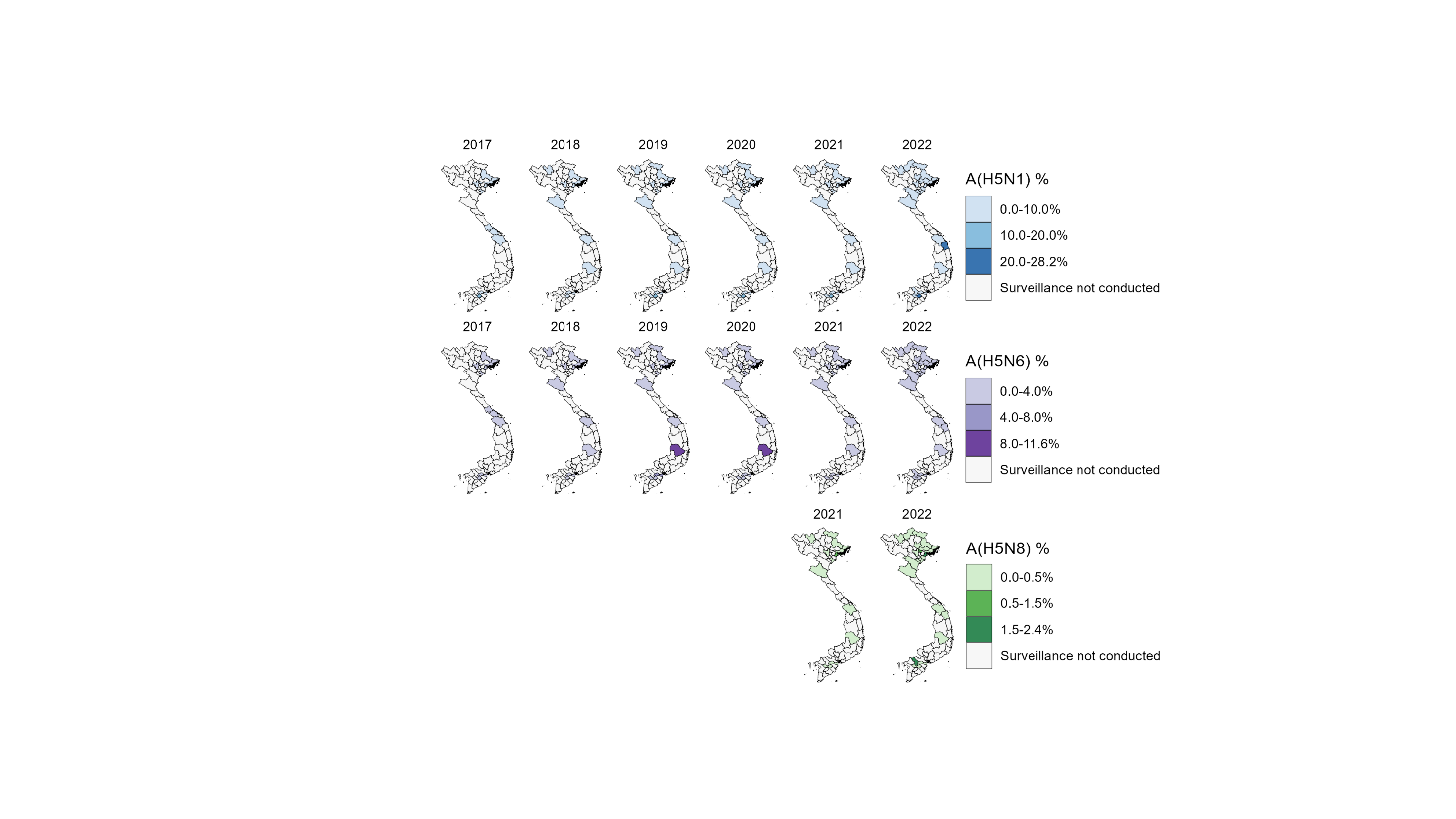
**

**Supplemental Figure 2. Influenza** **A(H5) sampling and test positivity from 2017–2022 across provinces that participated in active surveillance in live bird markets.** The number of samples each province tested for influenza A(H5) each month is indicated by the colored bars. The colors of the bars represent the region of Vietnam of each province where red indicates the North, orange Central, and yellow South. Monthly influenza A(H5) test positivity within each province is indicated by the black lines.


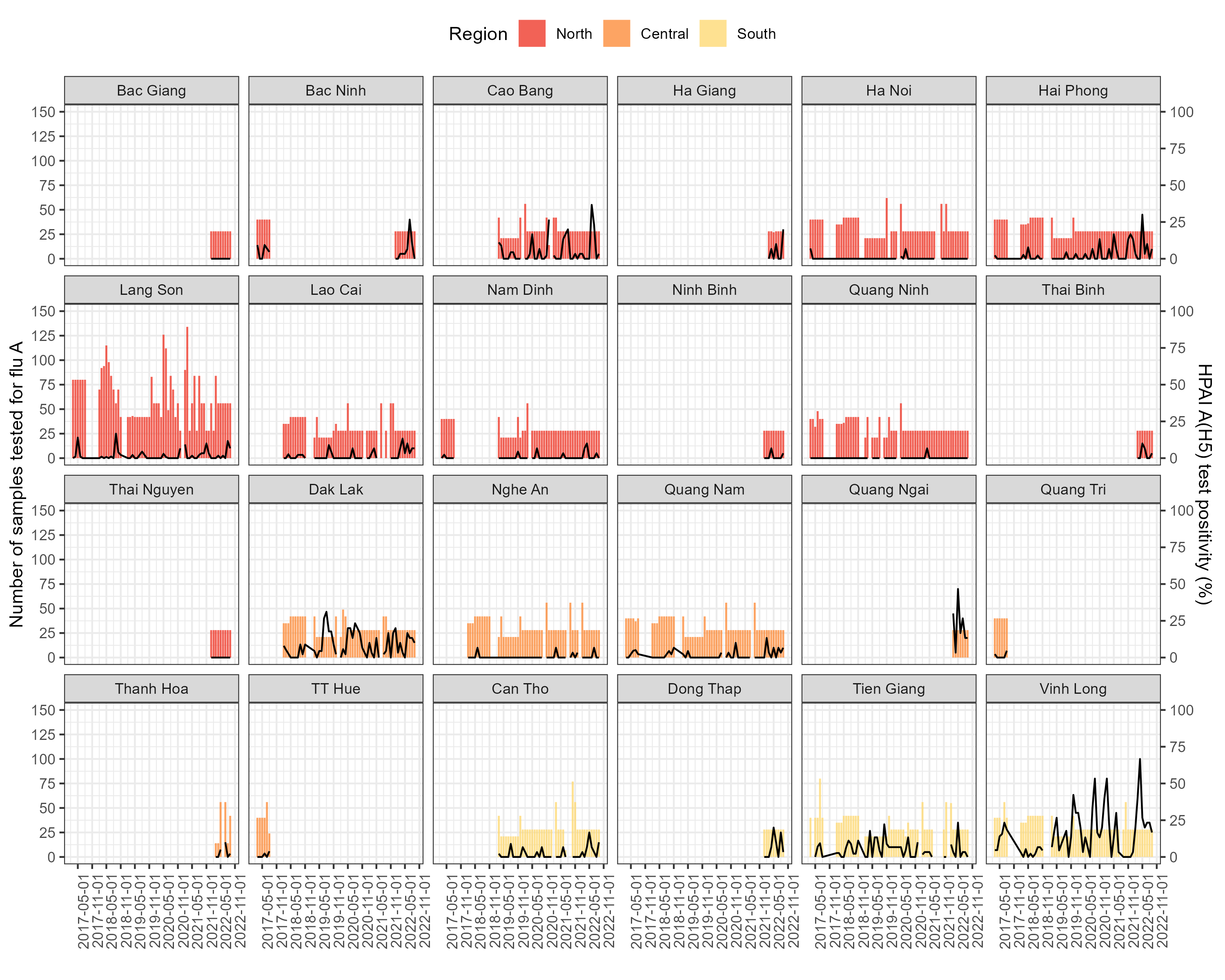

Supplement: Supplementary file 1 — Table S1. Prevalence of influenza A, A(H5), A(H5N1), A(H5N6), and A(H5N8) viruses by year at the live bird market, commune, district, and provincial levels. Table S2. Influenza A(H5) test positivity in 2022 across provinces included in active surveillance in live bird markets. Figure S1. Spatial distribution of influenza A(H5N1), A(H5N6), and A(H5N8) viruses detected in live bird markets. Provinces where surveillance was not conducted are shaded grey. Surveillance of A(H5N8) was not conducted from 2017–2020. Figure S2. Influenza A(H5) sampling and test positivity from 2017–2022 across provinces that participated in active surveillance in live bird markets. The number of samples each province tested for influenza A(H5) each month is indicated by the colored bars. The colors of the bars represent the region of Vietnam of each province where red indicates the North, orange Central, and yellow South. Monthly influenza A(H5) test positivity within each province is indicated by the black lines. [file IRV-17-e13245-s001.docx]
